# Supplementary material for: Impact of the COVID-19 Pandemic on Microbial Profiles and Clinical Outcomes in Orbital and Preseptal Cellulitis
Source: Microorganisms. 2024 Nov 8;12(11):2262. doi: 10.3390/microorganisms12112262 (PMC11596107; doi:10.3390/microorganisms12112262)
Supplement: Supplementary file 1 [file microorganisms-12-02262-s001.zip › Figure S2.pdf]

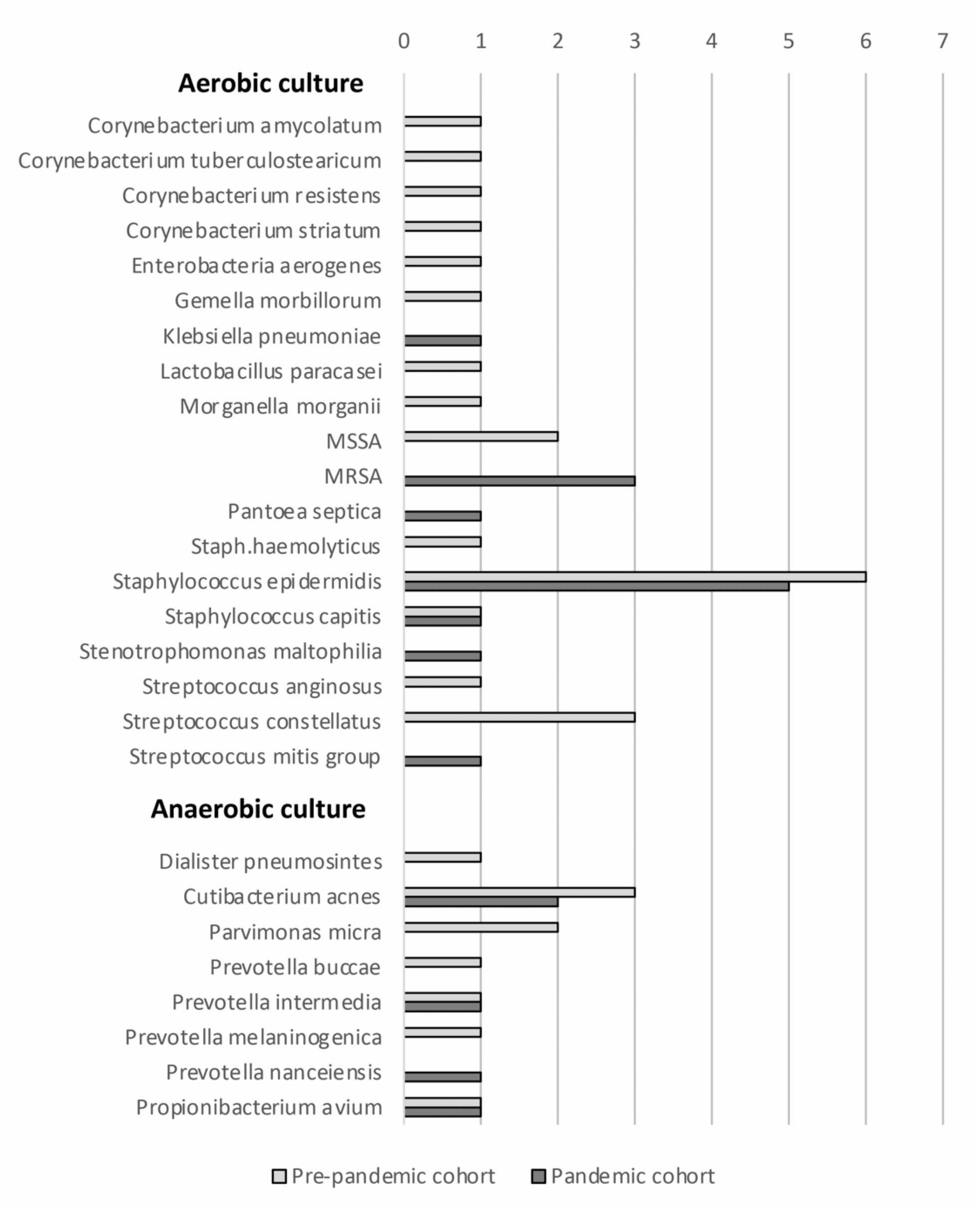


Figure S2: Identification of pathogens to the genus level isolated from surgical drainage cultures. MRSA = Methicillin-resistant Staphylococcus aureus; MSSA = Meticillin-sensitive Staphylococcus aureus; sp = Species
